# Exploring stress mediation: self-efficacy, sense of coherence, and self-esteem among nursing students in Poland—a multicenter cross-sectional observational study

**DOI:** 10.3389/fpubh.2025.1713759

**Published:** 2025-12-17

**Authors:** Anna Maria Cybulska, Ewa Kupcewicz, Kamila Rachubińska, Mariusz Panczyk, Anna Andruszkiewicz, Danuta Dyk, Aleksandra Gaworska-Krzemińska, Agnieszka Gniadek, Dorota Kozieł, Agnieszka Młynarska, Jolanta Lewko, Barbara Ślusarska, Magdalena Śniegocka, Elżbieta Grochans, Daria Schneider-Matyka

**Affiliations:** 1 Department of Nursing, Pomeranian Medical University in Szczecin, Szczecin, Poland; 2 Department of Nursing, Collegium Medicum, University of Warmia and Mazury in Olsztyn, Olsztyn, Poland; 3 Department of Education and Research of Health Sciences, Medical University of Warsaw, Warsaw, Poland; 4 Department of Basic Clinical Skills and Postgraduate Education of Nurses and Midwives, Nicolaus Copernicus University in Toruń, Bydgoszcz, Poland; 5 Department of Anaesthesiology and Intensive Care Nursing, Poznan University of Medical Sciences, Poznan, Poland; 6 Institute of Nursing and Midwifery, Faculty of Health Sciences with the Institute of Maritime and Tropical Medicine, Medical University of Gdańsk, Gdańsk, Poland; 7 Department of Nursing Management and Epidemiology, Nursing Institute of Nursing and Midwifery, Faculty of Health Sciences, Jagiellonian University Medical College, Kraków, Poland; 8 Jan Kochanowski University of Kielce Collegium Medicum, Kielce, Poland; 9 Department of Gerontology and Geriatric Nursing, School of Health Sciences, Medical University of Silesia, Katowice, Poland; 10 Department of Primary Health Care, Medical University Of Bialystok, Bialystok, Poland; 11 Department of Family and Geriatric Nursing, Chair of Integrated Nursing Care, Faculty of Health Sciences, Medical University of Lublin, Lublin, Poland; 12 Józef Piłsudski University of Physical Education in Warsaw, Warsaw, Poland

**Keywords:** nursing students, stress, sense of coherence, self-efficacy, self-esteem, mediation

## Abstract

**Background:**

Nursing students frequently encounter stressful and demanding situations during clinical training, which may hinder their well-being and professional development. A strong sense of coherence is thought to buffer the negative effects of stress and support adaptive coping.

**Objective:**

This study aimed to examine levels of sense of coherence, self-esteem, and self-efficacy among undergraduate nursing students in Poland, and to investigate whether perceived stress mediates the relationships between sense of coherence and self-esteem, as well as between sense of coherence and self-efficacy.

**Materials and methods:**

A cross-sectional observational survey was conducted among 2,689 nursing students enrolled in bachelor-level programs across multiple universities in Poland. Data collection utilized standardized tools, including the Sense of Coherence-29 Scale, the Generalized Self-Efficacy Scale, the Rosenberg Self-Esteem Scale, and the Perceived Stress Scale 10. Statistical analyses involved mediation modeling using Hayes’ PROCESS macro, with bootstrapping (5,000 resamples) to assess indirect effects.

**Results:**

Participants completed sociodemographic questionnaires along with standardized measures assessing sense of coherence, self-esteem, self-efficacy, and stress. The majority of students (84.0%) reported high stress levels, while two-thirds demonstrated average self-efficacy. Sex was associated with comprehensibility and manageability, and marital status was related to comprehensibility. Stress severity significantly mediated the relationships between specific components of sense of coherence and both self-esteem and self-efficacy.

**Conclusion:**

These findings emphasize the detrimental impact of stress on key components of sense of coherence and indicate that targeted educational and psychological interventions aimed at strengthening sense of coherence may help nursing students reduce stress, enhance self-efficacy, and promote overall well-being. The study underscores the multifaceted role of stress in shaping nursing students’ psychological resources and coping capacities.

## 1 Introduction

University education plays a key role in developing students’ self-esteem, assertiveness, and critical thinking (1). Nursing, one of the most demanding and stressful academic disciplines, requires extensive professional knowledge and competence (2–4). As a result, nursing students face numerous academic, clinical, and personal stressors (5) that can affect their well-being, performance, and quality of patient care (6, 7). Stress is understood as an interaction between the individual and the environment that exceeds personal resources and threatens well-being (8, 9). Difficulties in coping with educational challenges are common among nursing students (10). A strong sense of coherence serves as a protective factor against stress (11), supporting effective coping and promoting health through better resource utilization (12). It may also enhance students’ self-efficacy in learning (13).

Self-esteem is an emotional response to the difference between the ‘real self’ and the ‘ideal self’. It can include one dimension (value or competence), both dimensions, or multiple aspects related to academic sphere, family, or physical health. According to Rosenberg’s theory, self-esteem is the negative or positive attitude that individuals have of themselves. High self-esteem means having a positive and favorable view of oneself, while low self-esteem is characterized by a negative self-image and a kind of self-rejection (14). Self-esteem is a complex and multifaceted concept. High self-esteem is associated with greater motivation and better problem-solving. Low self-esteem, on the other hand, may be linked to mental disorders and a negative self-image, leading to a loss of self-respect (15).

It has been noted that the level of self-esteem among nursing students varies by country and culture. While some studies report that nursing students often have a high level of self-esteem (16, 17), others indicate that the level of self-esteem may be moderate or low (18, 19) and may also change during nursing education (20). The self-esteem of nursing students can affect many variables, such as well-being and mental health (21), quality of patient care (22), improving professional skills (23), professional identity of nursing graduates and their motivation (24). Self-esteem, on the other hand, may depend on year of study, as well as physical or mental health (25).

A review of the literature also indicates the significance of self-esteem as a moderator of stress (26). Additionally, it has been reported that nursing students with higher levels of stress are more likely to have lower self-esteem (4, 17). Since nursing students are exposed to various stressors during their education, it is important to measure their sense of coherence and identify its determinants. The sense of coherence consists of three components: *comprehensibility* (understanding of the world based on the ability to process familiar and unfamiliar stimuli in an orderly, structured, and coherent manner), *manageability* (how one deals with challenges and beliefs that challenges can be addressed with available resources), and *meaningfulness* (how an individual is motivated by constructing a meaningful life). A sense of coherence can support students in their education (13). However, there are no publications evaluating the relationship between the sense of coherence and global self-esteem or self-efficacy. Therefore, this study aimed to evaluate the levels of sense of coherence, global self-esteem, and self-efficacy among nursing students in Poland. In addition, stress was examined as a mediator between sense of coherence and global self-esteem, as well as between sense of coherence and self-efficacy. Based on these objectives, the following research hypotheses were formulated:

*H1*: There is a significant relationship between the level of self-efficacy and sense of coherence among nursing students.

*H2*: There are significant positive correlations between global self-esteem and sense of coherence among nursing students.

*H3*: There is a significant relationship in which stress severity mediates the association between sense of coherence and self-efficacy, as well as between sense of coherence and global self-esteem among nursing students.

## 2 Methods

### 2.1 Participants

This cross-sectional observational study was carried out at a single point in time in multiple universities across Poland offering undergraduate nursing programs. A total of 2,689 nursing students in the first degree (bachelor) studies in Poland were invited to participate in the study. The research was carried out from October to December 2023. This study is part of a larger project, part of which has been reported previously (27).

### 2.2 Sampling technique

A cross-sectional observational design was applied, and participants were recruited using a convenience sampling technique from multiple medical universities across Poland. Prior to data collection, permission to conduct the study was obtained from each participating institution.

### 2.3 Inclusion and exclusion criteria

The inclusion criteria for the study were:

- Being over 18 years of age,
- Current enrollment in a nursing program,
- Provision of informed consent to participate in the study, and
- No self-reported history of a diagnosed mental illness.

The size of the study sample was established on the basis of statistical data concerning the number of nursing students studying in Poland in the academic year 2023/2024. In the studied academic year, 36,909 people were studying nursing. The confidence level was set at 95%, the maximum error at 5%, and the estimated fraction size at 0.5. The minimum required sample size was calculated to be 381 students. The students who did not give informed consent to participate in the study were excluded. After obtaining permission from the dean’s authorities, trained researchers distributed paper versions of the questionnaires to nursing students.

The participants were informed about the purpose and anonymity of the study, and had the opportunity to ask questions and receive comprehensive explanations. They could opt out of the study at any time, without giving a reason. The time allotted to completing the questionnaire was about 15 min. A total of 3,000 sets of questionnaires were distributed, 2,689 of which were eligible for final analysis (return rate of 89.6%). The study is part of a larger project conducted among nursing students in Poland.

### 2.4 Data collection

This survey-based study was performed using a questionnaire technique. The following research tools were used to collect empirical data:

- The sociodemographic and academic proforma was used, which included questions on age, sex, year of study, and place of residence,
- Sense of Coherence Scale (SOC-29) is a 29-item instrument developed by Antonovsky (1983) to assess the overall level of the sense of coherence. It comprises three components: *Comprehensibility* (SOC-1), *Manageability* (SOC-2), and *Meaningfulness* (SOC-3). Each item is rated on a 7-point semantic differential scale, with total scores ranging from 29 to 203. The higher the score, the stronger the sense of coherence. The internal consistency coefficients, calculated using the split-half method with the Spearman–Brown correction, were 0.92 for the total sense of coherence, 0.78 for comprehensibility, 0.72 for manageability, and 0.68 for meaningfulness (28).
- Generalized Self-Efficacy Scale (GSES) is based on Bandura’s (1977, 1997) concept of self-efficacy expectations. It comprises 10 items, each rated on a 4-point Likert scale ranging from 1 (“not at all true”) to 4 (“exactly true”). Total scores range from 10 to 40, with higher scores reflecting greater perceived self-efficacy. The instrument demonstrates good internal consistency, with reported Cronbach’s alpha coefficients ranging from 0.76 to 0.90, most commonly around 0.80 (29).
- Rosenberg Self-Esteem Scale (RSES) consists of 10 statements assessing global self-esteem. Responses are given on a 4-point Likert scale (from 1 = “strongly disagree” to 4 = “strongly agree”). The total score may range from 10 to 40, with 10–25 indicating low self-esteem, 26–29 moderate, and 30–40 high self-esteem. The scale demonstrates good reliability, with Cronbach’s alpha between 0.81 and 0.83 (30).
- Perceived Stress Scale (PSS-10) is a 10-item instrument measuring the degree to which individuals perceive situations in their lives as stressful over the past month (Cohen et al., 1983). Each item is rated on a 5-point Likert scale (from 0 = “never” to 4 = “very often”). The total score may range from 0 to 40, with 0–13 indicating low stress, 14–26 moderate stress, and 27–40 high stress. The scale has demonstrated satisfactory internal consistency, with Cronbach’s alpha typically around 0.78 (31).

### 2.5 Data analysis

Mean (M), standard deviation (SD), median (Me), semi-interquartile range (IQR/2), as well as minimum and maximum values (Min. – Max.) were calculated for all continuous variables. Categorical variables were presented using frequencies and percentages.

To assess the variation in global self-esteem, stress intensity, among the study subgroups, the choice of statistical test was determined by the number of levels of the independent variable. Student’s t-test for independent samples was employed to compare the means between two groups, while one-way analysis of variance (ANOVA) was used to compare more than two groups.

The quantitative relationship between the independent variable and the dependent variable were analyzed using linear regression analysis in a mediation model to determine the mediating effect of stress on the relationship between sense of coherence and self-efficacy or global self-esteem. The mediation analysis was conducted using the method developed by Andrew F. Hayes (32). This approach was employed to determine whether stress mediates the relationship between sense of coherence and either self-efficacy or global self-esteem. The mediation model included the independent variable (X: sense of coherence), the mediator (M: stress), and the dependent variable (Y: self-efficacy or global self-esteem). To estimate the significance of the mediation effect, we used bootstrapping with 5,000 resamples to generate 95% confidence intervals for the indirect effect. In addition to calculating the significance of the mediation effect, we also computed the proportion of the total effect that was mediated. This was done by dividing the indirect effect by the total effect and multiplying by 100 to express it as a percentage. This allows us to quantify how much of the relationship between the independent variable and the dependent variable is explained by the mediator (33).

Statistical analyses were conducted using STATISTICA version 13.3 (TIBCO, Palo Alto, CA, USA). A significance level of *p* < 0.05 was adopted for all statistical tests, meaning that results were considered statistically significant if the probability of the observed differences or relationships occurring by chance was less than 5%.

### 2.6 Ethical considerations

The study was conducted in accordance with the Declaration of Helsinki after obtaining the approval of the Bioethics Committee of the (covered to blind review). The respondents were informed about the study’s purpose and could ask questions. They were assured of data confidentiality and their right to withdraw consent or participation at any time without consequences.

## 3 Results

### 3.1 Participants

The study involved 2,689 nursing students, including 2,460 women (91.48%) and 229 men (8.52%). The mean age was 21.1 years (SD = 2.1). Most respondents were single (64.71%), second-year nursing students (40.54%), studying at the Medical University of Silesia in Katowice (21.72%), and people living in a city with a population of more than 100,000 (39.42%) (Supplementary Table S1; General sociodemographic characteristics of the participants include their sex, place of residence, marital status, university, year of study, and age).

### 3.2 Description of stress, self-efficacy, sense of coherence, and global self-esteem scores of the participants

The assessment of stress intensity was aimed at identifying the participants’ subjective feelings related to personal problems and events, and ways of coping with them. Analysis showed that the mean sten score on the PSS-10 was 7.77 (SD = 1.31), and the mean raw result was 23.98 points (SD = 4.84). The mean sten score for sense of self-efficacy as measured by the GSES was 5.60 (SD = 1.18). The mean overall score for a sense of coherence according to the SOC-29 was 110 points (SD = 10.2), and the mean scores on the SOC-29 subscales were as follows: *comprehensibility* (SOC-1)—41.68 points (SD = 6.79), *manageability* (SOC-2)—37.51 points (SD = 4.94), and *meaningfulness* (SOC-3)—30.71 points (SD = 3.80). The mean result for global self-esteem according to the RSES was 25 points (SD = 2.17) [Supplementary Table S2; Descriptive statistics for the scales – presents descriptive statistics (mean, standard deviation, median, interquartile range, minimum, maximum, coefficient of variation) for the applied psychological scales (PSS-10, GSES, SOC-29, RSES)]. The results on the PSS-10, when converted into sten scores showed that 84.01% of the nursing students had high, 14.12% had average, and 1.79% had low levels of stress over the previous month. Furthermore, we found that 66.64% of the participants had average, 21.57% had high, and 11 0.79% had low sense of self-efficacy. The structure of the results on the PSS-10 and the GSES was shown in Supplementary Table S3. Psychological variables – shows the distribution of results in psychological scales categorized into low, average, and high sten scores.

### 3.3 Effects of sociodemographic variables on the sense of coherence

In the study, we analyzed the relationships between sociodemographic variables and a sense of coherence according to the SOC-29.

The analysis revealed a statistically significant link between sex and two components of coherence: *comprehensibility* (*p* < 0.001) and *manageability* (*p* = 0.013)-for both components, men scored higher than women. No statistically significant relationship was observed between sex and *meaningfulness* (Table 1).

**Table 1:** Effects of sociodemographic variables on the sense of coherence according to the SOC-29.

Variable		SOC-1 (Comprehensibility)	SOC-2 (Manageability)	SOC-3 (Meaningfulness)
Sex (M ± SD)	Women (*n* = 2,460)	41.55 ± 6.72	37.45 ± 4.89	30.66 ± 3.76
	Men (*n* = 229)	43.22 ± 7.50	38.30 ± 5.60	31.07 ± 4.37
	*t* (df = 2,687)	−3.565	−2.484	−1.564
	Cohen’s *d*	0.25	0.17	0.11
	95% CI	0.11–0.38	0.04–0.31	−0.03–0.24
	*p*-value*	<0.001	0.013	0.118
Marital status (M ± SD)	Single (*n* = 1740)	41.70 ± 6.72	37.70 ± 4.96	30.70 ± 3.77
	Married (*n* = 112)	43.80 ± 7.37	37.20 ± 4.53	30.10 ± 3.51
	Informal relationship (*n* = 837)	41.50 ± 6.87	37.30 ± 5.00	30.80 ± 3.96
	*F*	4.78	2.15	2.14
	*p*-value**	0.009	0.118	0.119
Year of study (M ± SD)	1st (*n* = 1,040)	41.60 ± 7.00	37.80 ± 5.05	30.90 ± 3.79
	2nd (*n* = 1,090)	41.60 ± 6.75	37.30 ± 4.89	30.60 ± 3.88
	3rd (*n* = 559)	42.10 ± 6.55	37.40 ± 4.92	30.50 ± 3.75
	*F*	1.31	2.00	1.90
	*p*-value**	0.271	0.136	0.149

* Student’s *t*-test. ** One-way ANOVA.

In addition, a statistically significant relationship was noted between marital status and *comprehensibility* (*p* = 0.009). A post-hoc test showed significant differences in this component between married and single individuals (*p* = 0.004) and those in an informal relationship (*p* = 0.003)-married subjects scored higher for this component than their single counterparts and those in an informal relationship. There were no statistically significant associations between marital status and *manageability* or *meaningfulness* (Table 1). There were no statistically significant relationships between coherence components and year of study (Table 1).

### 3.4 Relationships of the sense of coherence with the sense of self-efficacy among nursing students―a mediating role of stress

The study analyzed three mediation models to examine the role of perceived stress (assessed with the PSS-10) as a mediator of the relationships between the components of the sense of coherence: *comprehensibility* (SOC-1), *manageability* (SOC-2), and *meaningfulness* (SOC-3) and the sense of self-efficacy as measured by the GSES among nursing students.

Model 1 was used to assess whether stress severity (measured by the PSS-10) serves as an important mediator between *comprehensibility* (SOC-1) and the sense of self-efficacy according to the GSES. The results confirmed a statistically significant mediating effect of stress. The association between the sense of self-efficacy and the level of stress was examined. The indirect effect was negative and statistically significant (*b* = −0.01, 95% CI [−0.04, −0.01], *β* = −0.01, *z* = −6.45, *p* < 0.001), indicating that higher levels of *comprehensibility* were associated with lower stress, which in turn was related to greater self-efficacy. The direct effect of *comprehensibility* on self-efficacy was also statistically significant (*b* = 0.02, 95% CI [0.02, 0.03], *β* = 0.14, *z* = 6.18, *p* < 0.001), confirming partial mediation, with 23% of the total effect transmitted indirectly (Table 2).

**Table 2:** Indirect and total effects―mediation models 1, 2, 3.

Model	Effect type	Path	*b*	95% CI	*β*	*z*	*p*-value	Mediation (%)
Model 1	Indirect	SOC-1 ⇒ PSS-10 ⇒ GSES	−0.01	−0.04 – −0.01	-0.01	−6.45	<0.001	23.0
	Component	SOC-1 ⇒ PSS-10	−0.04	−0.05 – −0.03	−0.20	−10.02	<0.001	–
		PSS-10 ⇒ GSES	0.19	0.15–0.23	0.21	8.60	<0.001	–
	Direct	SOC-1 ⇒ GSES	0.02	0.02–0.03	0.14	6.18	<0.001	77.0
	Total	SOC-1 ⇒ GSES	0.02	0.01–0.03	0.10	4.52	<0.001	100.0
Model 2	Indirect	SOC-2 ⇒ PSS-10 ⇒ GSES	0.00	0.00–0.00	0.00	0.80	0.426	15.9
	Component	SOC-2 ⇒ PSS-10	0.00	−0.01 – 0.01	0.02	0.82	0.413	–
		PSS-10 ⇒ GSES	0.17	0.12–0.21	0.18	7.30	<0.001	–
	Direct	SOC-2 ⇒ GSES	0.00	−0.02 – 0.01	−0.02	−0.68	0.498	84.1
	Total	SOC-2 ⇒ GSES	0.00	−0.01 – 0.01	−0.01	−0.55	0.586	100.0
Model 3	Indirect	SOC-3 ⇒ PSS-10 ⇒ GSES	0.00	0.01–0.01	0.00	3.14	0.002	46.6
	Component	SOC-3 ⇒ PSS-10	0.03	0.01–0.04	0.08	3.64	<0.001	–
		PSS-10 ⇒ GSES	0.17	0.12–0.21	0.19	7.45	<0.001	–
	Direct	SOC-3 ⇒ GSES	−0.01	−0.02 – 0.01	−0.02	−0.64	0.519	53.4
	Total	SOC-3 ⇒ GSES	−0.000633	−0.02 – 0.01	0.00	−0.08	0.933	100.0

SOC-29, Sense of Coherence-29 scale; SOC-1, Comprehensibility; SOC-2, Manageability; SOC-3, Meaningfulness; GSES, Generalized Self-Efficacy Scale; PSS-10, Perceived Stress Scale-10.

Model 2 revealed that stress severity was not a mediator between *manageability* (SOC-2) and the sense of self-efficacy. The indirect effect was not statistically significant (*b* = 0.00, 95% CI [0.00, 0.00], *β* = 0.00, *z* = 0.80, *p* = 0.426). Neither the effect of *manageability* on the level of stress (*b* = 0.00, *p* = 0.413) nor its direct effect on self-efficacy (*b* = 0.00, *p* = 0.498) reached statistical significance. The total effect was likewise non-significant (*b* = 0.00, *p* = 0.586), indicating no mediation and no relationship between *manageability* and the sense of self-efficacy (Table 2).

Model 3, with *meaningfulness* (SOC-3) as the predictor, revealed a significant indirect effect on the sense of self-efficacy (*b* = 0.00, 95% CI [0.01, 0.01], *β* = 0.00, *z* = 3.14, *p* = 0.002). This finding indicates that higher levels of *meaningfulness* were associated with lower stress, which in turn translated into higher self-efficacy. However, the direct effect of *meaningfulness* on self-efficacy was not statistically significant (*b* = −0.01, *p* = 0.519), nor was the total effect (*b* ≈ 0.00, *p* = 0.933). These results suggest full mediation, with 46.6% of the total effect occurring indirectly, despite the overall effect not reaching statistical significance (Table 2).

Taken together, the analyses demonstrated that only Model 1 showed a significant partial mediation, suggesting that *comprehensibility*, as a component of the sense of coherence, reduces perceived stress levels, thereby enhancing the sense of self-efficacy as measured by the GSES. In contrast, Model 3 revealed a significant full mediation for *meaningfulness*, whereas Model 2 showed no significant effects.

### 3.5 Relationships of the sense of coherence with the global self-esteem among nursing students—a mediating role of stress

Analysis of mediation was also performed to assess whether stress severity (PSS-10) is an important mediator between the components of the sense of coherence: *comprehensibility* (SOC-1), *manageability* (SOC-2), and *meaningfulness* (SOC-3) and global self-esteem according to the RSES among nursing students. The results did not confirm statistically significant mediation.

The analysis (Model 4) did not reveal a significant indirect effect of *comprehensibility* (SOC-1) on global self-esteem (*b* = 0.00, 95% CI [0.00, 0.00], *β* = 0.00, z = 1.10, *p* = 0.272). Although the effect of *comprehensibility* on the level of stress was strong and negative (*b* = −0.04, *p* < 0.001), the effect of stress severity on global self-esteem (RSES) did not reach statistical significance (*b* = −0.04, *p* = 0.268). Conversely, the direct effect of *comprehensibility* on global self-esteem was positive and statistically significant (*b* = 0.06, 95% CI [0.04, 0.07], *β* = 0.17, *z* = 7.85, *p* < 0.001), accounting for 97.51% of the total effect (Table 3).

**Table 3:** Indirect and total effects―mediation model 4, 5, 6.

Model	Effect type	Path	*b*	95% CI	*β*	*z*	*p*-value	Mediation (%)
Model 4	Indirect	SOC-1 ⇒ PSS-10 ⇒ RSES	0.00	0.00–0.00	0.00	1.10	0.272	2.49
	Component	SOC-1 ⇒ PSS-10	−0.04	−0.05 – −0.03	−0.20	−9.58	<0.001	–
		PSS-10 ⇒ RSES	−0.04	−0.10 – 0.03	−0.02	−1.11	0.268	–
	Direct	SOC-1 ⇒ RSES	0.06	0.04–0.07	0.17	7.85	<0.001	97.51
	Total	SOC-1 ⇒ RSES	0.06	0.04–0.07	0.18	7.96	<0.001	100.0
Model 5	Indirect	SOC-2 ⇒ PSS-10 ⇒ RSES	−0.000418	0.00–0.00	0.00	−0.74	0.457	0.98
	Component	SOC-2 ⇒ PSS-10	0.00	−0.01 – 0.02	0.02	0.81	0.417	–
		PSS-10 ⇒ RSES	−0.09	−0.16 – −0.03	−0.06	−2.85	0.004	–
	Direct	SOC-2 ⇒ RSES	−0.04	−0.06 – −0.02	−0.10	−4.38	<0.001	99.02
	Total	SOC-2 ⇒ RSES	−0.04	−0.06 – −0.02	−0.10	−4.43	<0.001	100.0
Model 6	Indirect	SOC-3 ⇒ PSS-10 ⇒ RSES	0.00	−0.000471 – 0.00	0.00	−2.20	0.028	9.53
	Component	SOC-3 ⇒ PSS-10	0.03	0.01–0.04	0.08	3.70	<0.001	–
		PSS-10 ⇒ RSES	−0.09	−0.15 – −0.02	−0.05	−2.69	0.007	–
	Direct	SOC-3 ⇒ RSES	−0.02	−0.05 – 0.00	−0.04	−1.87	0.061	90.47
	Total	SOC-3 ⇒ RSES	−0.02	−0.05 – −0.0000356	−0.04	−2.10	0.036	100.0

SOC-29, Sense of Coherence-29 scale; SOC-1, Comprehensibility; SOC-2, Manageability; SOC-3, Meaningfulness; RSES, Rosenberg Self-Esteem Scale; PSS-10, Perceived Stress Scale-10.

Focused on *manageability* (SOC-2), Model 5 showed that stress severity does not mediate the relationship between this component and global self-esteem (*β* = 0.00, *z* = −0.74, *p* = 0.457). Although perceived stress had a significant effect on global self-esteem (RSES) (*b* = −0.09, 95% CI [−0.16, −0.03], *β* = −0.06, *z* = −2.85, *p* = 0.004), *manageability* did not significantly predict perceived stress (*b* = 0.00, *p* = 0.417). The direct effect of *manageability* on global self-esteem was negative and significant (*b* = −0.04, 95% CI [−0.06, −0.02], *β* = −0.10, *z* = −4.38, *p* < 0.001), indicating a direct negative influence of *manageability* on global self-esteem. Mediation was negligible, accounting for only 0.98% of the total effect (Table 3).

Model 6 showed that stress severity is a mediator between *meaningfulness* (SOC-3) and global self-esteem (RSES) (*β* = 0.00, *z* = −2.20, *p* = 0.028). *Meaningfulness* significantly predicted lower levels of perceived stress (*b* = 0.03, *p* < 0.001). Higher perceived stress, in turn, was associated with lower global self-esteem (*b* = −0.09, *p* = 0.007). The direct effect of *meaningfulness* on global self-esteem was close to statistical significance (*b* = −0.02, 95% CI [−0.05, 0.00], *β* = −0.04, *z* = −1.87, *p* = 0.061), whereas the total effect was statistically significant (*b* = −0.02, *p* = 0.036), indicating a partial but weak mediation of perceived stress, accounting for 9.53% of the total effect (Table 3).

In summary, none of the models (4–6) revealed a strong mediating effect of perceived stress in the relationship between the sense of coherence components and global self-esteem. Models 4 and 5 demonstrated the predominance of the direct effect (positive and negative, respectively), whereas Model 6 indicated a weak, partial mediation of perceived stress for *meaningfulness* (SOC-3).

## 4 Discussion

### 4.1 The level of stress among nursing students

In the present study, most nursing students experienced high stress and an average sense of self-efficacy (4). Similar results were reported by Kupcewicz et al. (34) and in the meta-analysis by Zheng et al. (34), both indicating moderate to high stress among nursing students. Lavoie-Tremblay et al. (4) observed that stress was particularly high among first-year students, while Hasimi et al. (35) found that low coherence and resilience were predictors of psychological distress. Other studies have also highlighted high rates of anxiety, depression, and overall psychological strain among nursing students, underscoring the need for continuous monitoring and preventive interventions (36).

In this study, the mean PSS-10 score among participants was 23.98 ± 4.84 points. Various authors claim that students in other fields of study experience stress at a similar or much higher level than in our own research. Alsaleem et al. (37), for example, noted the average PSS-10 score of 19.13 ± 6.56. Lower scores were obtained among students studying at the University of India (19.2 points) (37), Nigeria (19.6 points) (38), Iran (20.04 points) (39), France (15.9 points) (40), USA (16 points) (41) or England (17.9 points) (42). In contrast, higher scores for perceived stress were recorded among students in Malaysia (27.5 points) (43) and Riyadh (27.0 points) (44). A wide variation in the levels of stress may be related to differences in study design and differences in the severity of personal, family, financial, and academic stressors.

### 4.2 Impact of sociodemographic variables on the sense of coherence

The present study demonstrated a statistically significant association between sex and the sense of coherence, specifically the components of *comprehensibility* and *manageability*, with men achieving higher scores than women. Moreover, a statistically significant relationship was observed between marital status and *comprehensibility*. People in a formal relationship scored higher for this component of coherence than those who were single or in an informal relationship.

Antonovsky believes that the sense of coherence depends on one’s life experiences as well as sex, age, culture, ethnicity, and genetic factors (45). A review of the literature indicates that sex is one of the main factors that shape the sense of coherence (46–49). However, there is no conclusive data on who have higher sense of coherence―women or men. Based on a systematic review, Rivera et al. (50) concluded that in most studies, it was men who scored higher for sense of coherence than women. Dietscher et al., on the other hand, assert that both sex and socioeconomic status play an important role in the development of coherence (51).

Hasimi et al. (35) found no significant difference in the total score or any of the dimensions of coherence depending on age, sex, marital status, or year of study.

### 4.3 Relationship of the sense of coherence with the sense of self-efficacy and global self-esteem of nursing students―the mediating role of stress

A review of the literature indicates that there are few studies evaluating the role of stress as mediator between the sense of coherence and the sense of self-efficacy or global self-esteem among nursing students. Therefore, these findings help to fill an important information gap in this field. The results indicate that the severity of stress acts as a partial mediator between the sense of coherence in the domain of *comprehensibility* and the sense of self-efficacy. Moreover, stress severity was found to be negatively associated with the sense of coherence in the domain of *comprehensibility*, and positively associated with the domain of *manageability*. The finding indicating a direct negative influence of manageability on global self-esteem may arise from several factors. First, students with a high sense of manageability may feel a greater responsibility to cope effectively with challenges, which can lead to increased self-criticism when difficulties occur, ultimately lowering their global self-esteem. Second, nursing students frequently operate in highly demanding environments, where the ability to manage stressors does not necessarily translate into positive self-evaluation. This mismatch may contribute to reduced self-esteem. Another possibility is that high manageability reflects a control-focused coping style which, under conditions of elevated stress, may become overwhelmed, leading to frustration and diminished self-worth.

Kupcewicz (52) found that the general sense of coherence partially mediates the relationship between global self-esteem and the severity of perceived stress. The study also demonstrated that global self-esteem (independent variable) was significantly correlated with the general sense of coherence (mediator), even when the dependent variable perceived stress severity was excluded. Moreover, the higher the regression coefficient between global self-esteem and stress severity, the stronger the likelihood of a causal relationship. When analyzing the components of sense of coherence, it was shown that all three components *comprehensibility*, *manageability*, and *meaningfulness* were significantly associated with perceived stress.

Similarly, Hasimi et al. (35) reported negative correlations between the General Health Questionnaire (GHQ) scores, resilience, and sense of coherence (SOC), indicating that students with higher resilience and stronger SOC experienced better mental health. Linear regression analysis further confirmed that both SOC and resilience were significant predictors of mental health among nursing students. Consistent findings were obtained by Eriksson et al. (53), who confirmed that a strong sense of coherence was linked to better self-perceived mental health. Conversely, low sense of coherence increases vulnerability to psychological distress, as individuals with a weaker sense of coherence tend to experience greater stress in everyday life and possess fewer effective coping mechanisms.

Wu et al. (54) also highlighted the mediating role of coherence in the relationship between stress/resource complex subtypes and professional identity among nursing students. Their study indicated that a limited perception of problems (low *comprehensibility*) may hinder students’ ability to recognize available internal and external resources (*manageability*), thereby impairing effective coping with professional challenges.

Overall, the three components of Antonovsky’s model—*comprehensibility*, *manageability*, and *meaningfulness*—constitute key protective factors for mental health. They should therefore be integrated into interventions aimed at strengthening psychological resilience and well-being among nursing students (45). A review of the literature further supports that a high sense of coherence is associated with greater psychological resources, stronger motivation and engagement, and healthier behavioral patterns (55–57).

## 5 Limitations

The study has some limitations. It only involved nursing students from selected medical universities in Poland. The research was carried out at the beginning of the semester before the exams began, so it can be assumed that the pressure to pass the exams was reduced.

One of the strengths of our study is the large number of participants from different parts of Poland, which allows us to obtain meaningful results and to create a support program for nursing students.

## 6 Conclusion

- The vast majority of students experienced high levels of stress and demonstrated average self-efficacy. Therefore, it appears essential to develop curricula that strengthen students’ sense of coherence and self-esteem, as well as support the adoption of effective stress-coping strategies.
- Sex and marital status are important factors affecting the sense of coherence.
- Stress severity serves as a mediator between selected components of sense of coherence and self-efficacy among nursing students. Stress was shown to diminish the sense of coherence, particularly in the components of *comprehensibility* and *manageability*.

### 6.1 Relevance for clinical practice

The findings provide valuable insights that can be translated into strategies supporting the education and well-being of nursing students. Most participants reported high levels of stress and moderate self-efficacy, highlighting the need to incorporate stress management components into nursing curricula. Implementing workshops aimed at building psychological resilience and coping skills may enhance students’ ability to manage academic and clinical challenges more effectively, both during their studies and in future professional practice.

Strong sense of coherence and positive self-esteem are essential for maintaining mental health and motivation among nursing students. Therefore, integrating activities that promote these attributes such as motivational strategies, self-confidence training, and reflective practice could further improve students’ well-being and learning outcomes. In addition, providing mentors and academic staff with training to recognize signs of student stress and to offer appropriate psychological support could improve the overall educational experience. Future research should consider longitudinal follow-up studies to evaluate the long-term impact of such interventions, as well as experimental approaches to assess the effectiveness of specific educational and psychosocial programs aimed at strengthening resilience and self-efficacy among nursing students. In conclusion, implementing structured psychological support and resilience-building programs within nursing education is crucial for enhancing the quality of education, promoting student well-being, and ultimately improving patient care outcomes.

## Data Availability

The raw data supporting the conclusions of this article will be made available by the authors, without undue reservation.
