# Supplementary material for: Exploring stress mediation: self-efficacy, sense of coherence, and self-esteem among nursing students in Poland—a multicenter cross-sectional observational study
Source: Front Public Health. 2025 Dec 17;13:1713759. doi: 10.3389/fpubh.2025.1713759 (PMC12753474; doi:10.3389/fpubh.2025.1713759)
Supplement: Supplementary file 1 [file Table_1.DOCX]

***Supplementary:***

Table 1. General sociodemographic characteristics of the study group

| **Variable** | | **n** | **%** |
| --- | --- | --- | --- |
| **Sex** | woman | 2460 | 91.48 |
|  | man | 229 | 8.52 |
| **Place of residence** | rural areas | 933 | 34.70 |
|  | city with a population of 10,000–100,000 | 696 | 25.88 |
|  | city with a population of over 100,000 | 1060 | 39.42 |
| **Marital status** | single | 1740 | 64.71 |
|  | informal relationship | 837 | 31.13 |
|  | married | 112 | 4.17 |
| **University** | Medical University of Silesia in Katowice | 584 | 21.72 |
|  | Jagiellonian University―Medical College | 314 | 11.68 |
|  | Medical University of Lublin | 275 | 10.23 |
|  | Nicolaus Copernicus University in Toruń―Ludwik Rydygier Collegium Medicum in Bydgoszcz | 249 | 9.26 |
|  | Pomeranian Medical University in Szczecin | 217 | 8.07 |
|  | Medical University of Białystok | 196 | 7.29 |
|  | Jan Kochanowski University (Kielce) | 177 | 6.58 |
|  | Józef Piłsudski University of Physical Education in Warsaw | 173 | 6.43 |
|  | Medical University of Gdańsk | 158 | 5.88 |
|  | Poznań University of Medical Sciences | 142 | 5.28 |
|  | University of Warmia and Mazury in Olsztyn | 110 | 4.09 |
|  | Higher School of Strategic Planning in Dąbrowa Górnicza | 94 | 3.50 |
| **Year of study** | 1 | 1040 | 38.68 |
|  | 2 | 1090 | 40.54 |
|  | 3 | 559 | 20.79 |
| **Age** | ≤ 20 | 1161 | 43.18 |
|  | 21–25 | 1428 | 53.11 |
|  | 26–30 | 90 | 3.35 |
|  | > 30 | 10 | 0.04 |

Table 2. Descriptive statistics for the scales

| **Variable** | | **M** | **SD** | **Mdn** | **IQR/2** | **Min** | **Max** | **CV [%]** |
| --- | --- | --- | --- | --- | --- | --- | --- | --- |
| PSS-10 | sten scores | 7.77 | 1.31 | 8.0 | 1.00 | 1 | 10 | 16.87 |
|  | points | 23.98 | 4.84 | 24.0 | 6.00 | 0 | 40 | 20.20 |
| GSES | sten scores | 5.60 | 1.18 | 6.0 | 0.50 | 1 | 10 | 21.04 |
| SOC-29 | total | 110 | 10.2 | 110 | 12.0 | 43 | 203 | 9.3 |
|  | SOC-1 (*Comprehensibility)* | 41.68 | 6.79 | 42.0 | 4.50 | 18 | 77 | 16.28 |
|  | SOC-2 (*Manageability)* | 37.51 | 4.94 | 37.0 | 3.00 | 15 | 70 | 13.16 |
|  | SOC-3 (*Meaningfulness)* | 30.71 | 3.80 | 31.0 | 2.50 | 9 | 56 | 12.38 |
| RSES | points | 25.00 | 2.17 | 25.0 | 1.00 | 15 | 42 | 8.69 |
| M*―*mean, SD*―*standard deviation, Mdn*―*median, IQR/2*―*interquartile range, Min*―*minimum, Max*―*maximum, CV*―*coefficient of variation | | | | | | | | |

**Table 3. Psychological variables**

| **Variable** | | **n** | **%** |
| --- | --- | --- | --- |
| **PSS-10 (sten score)** | low results (sten score of 1–4) | 48 | 1.79 |
|  | average results (sten score of 5–6) | 382 | 14.21 |
|  | high results (sten score of 7–10) | 2259 | 84.01 |
| **GSES (sten score)** | low results (sten score of 1–4) | 317 | 11 .79 |
|  | average results (sten score of 5–6) | 1792 | 66 .64 |
|  | high results (sten score of 7–10) | 580 | 21 .57 |
